# Investigation of the Influence of Pulse Duration and Application Mode on Microsecond Laser Microsurgery of the Retinal Pigment Epithelium

**DOI:** 10.3390/life13061314

**Published:** 2023-06-02

**Authors:** Christian Burri, Simon Salzmann, Mylène Amstutz, Leonie Hoffmann, Boris Považay, Christoph Meier, Martin Frenz

**Affiliations:** 1 Biomedical Photonics Group, University of Bern, Sidlerstrasse 5, 3012 Bern, Switzerland; martin.frenz@iap.unibe.ch; 2 Institute for Human Centered Engineering (HuCE)—OptoLab, Bern University of Applied Sciences, Quellgasse 21, 2501 Biel, Switzerland; simon.salzmann@bfh.ch (S.S.); mylene.amstutz@bfh.ch (M.A.); leonie.hoffmann@bfh.ch (L.H.); boris.povazay@bfh.ch (B.P.); christoph.meier@bfh.ch (C.M.)

**Keywords:** selective retina therapy, real-time feedback-controlled dosimetry, micropulse laser, RPE rejuvenation, microbubble formation, photodisruptive laser therapy, fringe washout, RELITE

## Abstract

**Featured Application:**

**Real-time feedback-controlled dosimetry of selective retina therapy (SRT) using spectral domain optical coherence tomography. SRT is a minimally invasive cellular-level surgical laser treatment method for various diseases of the fundus, i.e., central serous chorioretinopathy or diabetic macular edema, associated with reduced retinal pigment epithelium function.**

**Abstract:**

Optical microsurgery confined to the retinal pigment epithelium (RPE) requires locally optimized laser parameters and reliable real-time feedback dosimetry (RFD) to prevent unwanted neuroretinal overexposure. This study aimed to compare pulses of different durations and application modes (single, ramp, burst). Moreover, optical coherence tomography (OCT)-based RFD was investigated in an ex vivo experiment, utilizing nine porcine eyes that were exposed to laser pulses of 8, 12, 16 and 20 µs duration (wavelength: 532 nm, exposure area: 90 × 90 µm^2^, radiant exposure: 247 to 1975 mJ/µm^2^). Simultaneously, time-resolved OCT M-scans were recorded (central wavelength: 870 nm, scan rate: 85 kHz) for RFD. Post irradiation, retinal changes were assessed with color fundus photography (CFP) and cross-sectional OCT B-scans. RPE cell damage was quantified via fluorescence-based cell viability assay and compared to the OCT dosimetry feedback. Our experiments indicate cumulative RPE damage for pulse bursts of 16 µs and 20 µs, whereas no cumulative effects were found for pulse durations of 8 µs and 12 µs applied in ramp mode. According to statistical analysis, OCT-RFD correctly detected RPE cell damage with 96% sensitivity and 97% specificity using pulses of 8 µs duration in ramp mode.

## 1. Introduction

For decades, laser applications for the treatment of retinal diseases have been pursued due to their elegance of direct access through the ocular media and the possibility of sub-millimeter precise positioning under visual guidance. For retinal diseases, the retinal pigment epithelium (RPE), a monolayer of typically post-mitotic polarized epithelial cells strategically situated between the photoreceptors and the choroid, plays an important role as the primary caretaker of photoreceptor health and function [1]. RPE dysfunction apparently underlies many inherited and acquired diseases causing permanent blindness [2]. The resilience and regenerative capacity of the RPE has prompted the development of several laser techniques aimed at stimulating and rejuvenating the RPE whilst staying within a treatment window beneath the thresholds for collateral damage. To best meet the requirements for RPE-specific treatment, a photodisruptive laser approach known as selective retina therapy (SRT) was found to be particularly suitable [3]. SRT intends to selectively initiate apoptosis of dysfunctional or senescent patches of the approximately 10 μm thick RPE monolayer without scarring the choroid and the neurosensory retina and especially sparing the non-regenerative photoreceptors, to enable RPE-mitosis, regrowth and uptake of physiological retinal function [1,3,4,5]. The basis for selective damage is the RPE’s high melanin content in the intracellular melanosomes, which absorb about 50% of the incident light in the green spectral range in their function as stray-light suppressors [1,6]. Within the thermal confinement of the short microsecond-long pulses used for SRT, peak temperatures (140 to 150 °C) at the melanosomes in the RPE initiate microsecond-long microbubble formation (MBF) [7,8]. Rapid mechanical expansion and collapse of these micrometer-sized steam bubbles successively cause RPE cell wall disruption, followed by immediate or delayed cell death [8].

The original idea for selective RPE photocoagulation was introduced by Roider and Birngruber [4,9] following the concept of selective photothermolysis of the RPE by Anderson and Parish [10]. Subsequently, the concept was further developed and optimized by Brinkmann et al. at the Medical Laser Center Lübeck, and finally named SRT [11]. In contrast to conventional photocoagulation, SRT intends to rejuvenate the regenerative RPE, resulting in improved metabolism at the target sites after RPE migration and proliferation [3,12]. Since the concept of SRT was introduced, the method has been successfully used clinically to treat central serous chorioretinopathy and diabetic macular edema [11,13,14,15,16]. Furthermore, SRT has already been shown to reduce Bruch’s membrane thickness and age-related macular degeneration (AMD)-like RPE alterations in AMD mouse models to increase permeability and could therefore become a promising option for the treatment of dry AMD [17].

Recently, new high-power flexible continuous wave (CW)-based lasers have been investigated for SRT, allowing a wider range of pulse durations. For example, Seifert et al. evaluated a 15 W diode laser (A.R.C Laser, Nürnberg, Germany) at 514 nm wavelength and adjustable pulse duration from 2 to 50 µs [18], and Burri et al. evaluated a 30 W optically pumped semiconductor laser (Meridian Medical, Thun, Switzerland) at 532 nm and adjustable pulse duration from 2 µs to 20 µs [19]. Such lasers have the potential to cover the entire laser treatment range from SRT to sublethal hyperthermia-inducing short pulse applications to traditional CW photocoagulation. Since thermally induced tissue alterations depend on pulse duration, radiant exposure, and local temperature rise as well as tissue properties, effects differ widely among the application regimes, as do their medical indications. Common CW lasers used in clinical practice for photocoagulation operate in the range of a few hundred milliwatts to several watts of power. A laser modulated for SRT that is only active with a significantly lower duty cycle, on the other hand, requires higher instantaneous power to deliver the same amount of energy. As indicated by Seifert et al., who extrapolated clinical data for SRT on patients with diabetic macular edema, laser powers of 5 W to 40 W are required to achieve RPE damage using short microsecond laser pulses [18]. Although immunohistochemical studies by Framme et al. have already demonstrated selective RPE interaction with exposure times up to 15 µs [20], it is also known that due to the theory-derived thermal relaxation time for RPE cells, pulse durations longer than 10 µs may be less efficient for SRT [3]. Lee et al. substantiated this theoretical assumption when they showed that, in the case of 1, 5 and 10 µs laser pulse duration, more than 95% of dead RPE cells were associated with MBF, whereas the ratio decreased to 65% and 45% for longer 20 µs and 40 µs pulses, respectively [21]. Complementarily, an in vivo experiment by Burri et al. showed that for pulse durations of 20 µs, more pronounced morphologic retinal changes occur according to optical coherence tomography (OCT) B-scans compared to pulse durations of 4 µs [22]. It can be concluded that the pulse duration range of around 10 µs up to 20 µs, respectively, has to be carefully evaluated to achieve the desired effect according to SRT, but at the same time to avoid suprathreshold lesions with unwanted collateral effects to ensure clinical safety. Therefore, we examined the influence of pulse duration and application mode on laser microsurgery of the RPE in the range from 8 µs to 20 µs to investigate and thereby minimize the risk of heat accumulation in the neurosensory retina during SRT.

Another aspect we investigated in this study is real-time feedback dosimetry (RFD) for SRT. Even if basic parameters such as the laser pulse duration and the number of applied pulses as well as the pulse energy range are optimized, SRT without damaging the surrounding tissue by heat diffusion proved to be challenging. The reason for this is the strongly varying RPE melanin concentration within different locations by up to a factor of 4 [23], as well as the decreasing ocular transparency with age by tens of percent [24,25]. This leads to strongly varying inter- and intraindividual thresholds for selective RPE cell damage. Therefore, reliable RFD is essential to preserve photoreceptor integrity. Hence, several approaches for real-time MBF detection are under development. Delivering real-time feedback on the localized impact is imperative for keeping the laser energy within the treatment window, at best without additional mechanical interaction to minimize the procedure’s invasiveness. Methods such as measuring the increased reflectance at the bubble surface via backscattered light or capturing the ultrasonic emission caused by rapid bubble expansion and collapse to detect the presence of MBF have been successfully tested [26,27]. An attractive dosimetry approach is the utilization of spectral-domain optical coherence tomography (SD-OCT) simultaneously with SRT. This method was first described by Steiner et al., who indirectly detected tissue effects of laser pulses as signal loss (coherent fringe washout) in time-resolved SD-OCT A-scans (M-scans) [28,29,30]. The detailed mechano-optical model of the signal loss during laser treatment is still debated, but the currently favored hypothesis explains it as interference signal contrast loss, or decorrelation, also known as “fringe washout” resulting from the axial motion or other signal alterations of retinal surfaces due to MBF within time scales close to, or above, the coherent acquisition time of the depth scan [31]. In consideration are fast dynamic changes of the scattering behavior, especially at the level of the RPE as well as vibration due to MBF and thermal expansion following the exposure. Thereby, fringe washouts are particularly pronounced during a single A-scan (within the acquisition time of the interferometric measurement of typically 10 µs to 30 µs), which roughly corresponds to the average cumulative microsecond lifetime of MBF during SRT [8]. Burri et al. recently presented an SD-OCT-based RFD algorithm for SRT [31]. For certain samples, this algorithm achieved a sensitivity of 99% and specificity of 97% for predicting RPE lesions after microsecond laser application. We used the same algorithm again in this study to investigate OCT-based RFD.

## 2. Materials and Methods

### 2.1. Treatment and Monitoring System

For the experiments, the non-commercially available research device Spectralis Centaurus (HuCE-optoLab, Bern University of Applied Sciences, Biel, Switzerland) was used [32]. The device is based on a modified diagnostic imaging platform (Spectralis, Heidelberg Engineering, Heidelberg, Germany), extended with a prototype treatment laser (modified Merilas 532 short pulse, Meridian Medical, Thun, Switzerland) intended for SRT. The laser emits radiation at 532 nm, supports pulse durations from 2 to 20 μs at repetition rates of 100 Hz, and delivers 30 W power [19]. The laser beam is focused to a spot size of approximately 90 × 90 μm^2^ as a square beam profile on the porcine retina and an intensity modulation factor (IMF) of 1.3 is achieved, indicating an almost homogeneous top-hat beam profile [33]. The IMF describes the ratio of maximum to mean radiant exposure across the beam profile and was first introduced by Framme et al. in 2002 as a speckle factor [34]. An IMF = 1 corresponds to perfectly homogenous, top-hat radiant exposure. Furthermore, the system features the ability of intervention planning utilizing a coaxially integrated confocal scanning laser ophthalmoscope (cSLO) and a spectral domain (SD)—also called Fourier-domain (FD) OCT—that are widely applied in the diagnosis of retinal diseases by capturing cross-sectional and volumetric images (B- and C-scans). The super-luminescence diode of the SD-OCT laser emits infrared radiation centered at 870 nm wavelength with a 73 nm spectral bandwidth. The OCT system is operated at a rate of 85 kHz resulting in an integration time of 11.8 µs per A-scan. In B-scan mode, the beam scans across the retina, producing a cross-sectional, depth-resolved image of the backscatter from minute tissue interfaces. Furthermore, it can be operated in the so-called M-scan mode (motion mode) to measure time-resolved sequences of A-scans at the point of the therapy laser application, thereby unveiling depth-resolved temporal signal fluctuations or signal loss (coherent fringe washouts) for RFD.

### 2.2. Tissue Preparation

Juvenile porcine eyes were used for the experiments (*n* = 9). The enucleated pig eyes were obtained from a local slaughterhouse (pigs’ age ~24 weeks, ~110 kg weight). After enucleation, the eyes were transported to the laboratory (within 30 min) in a jar filled with phosphate-buffered saline (PBS), enclosed by a thermo box cooled with reusable ice packs (freshly enucleated eyes should be kept at 4 °C [35]). Before treatment, superfluous tissue was removed from around the eyeball. For irradiation, the eyes were positioned in a special 3D-printed plastic container (polylactic acid (PLA), Ultimaker S5, Utrecht, The Netherlands) attached to the original headrest in front of the delivery optics of the treatment system. To maintain the intraocular pressure (IOP), an intravitreal injection into the vitreous cavity through the pars plana was made. The eyes were cannulated using a disposable hypodermic needle (20 G × 1 1/2″, Teqler, Wecker, Luxembourg) connected with a tube to a height-adjustable column filled with balanced salt solution (BSS). A physiological pressure transducer (SP844, Memscap, Isere, France), inserted in the silicon tube at the same height as the eye via an attached silicon diaphragm dome (844-28, Memscap, Isere, France), allowed the recording, respectively, the control of the IOP. The eyes were pressurized at 15 mmHg. This corresponds roughly to the IOP found in pigs (15.2 mmHg) [36] and also to the mean IOP of humans (14.7 mmHg) [37]. The cornea was manually moistened using BSS approximately 10 times per minute. An intact corneal tear film is important for maintaining epithelial integrity, physiologically reduces surface scattering, and helps to partially correct high-order optical aberrations by smoothing out the rough ocular surface [38]. The experiments took place at room temperature well within the common cellular survivability window, which should not be longer than five hours [35].

### 2.3. Treatment Pattern and Irradiation

**Figure 1:**
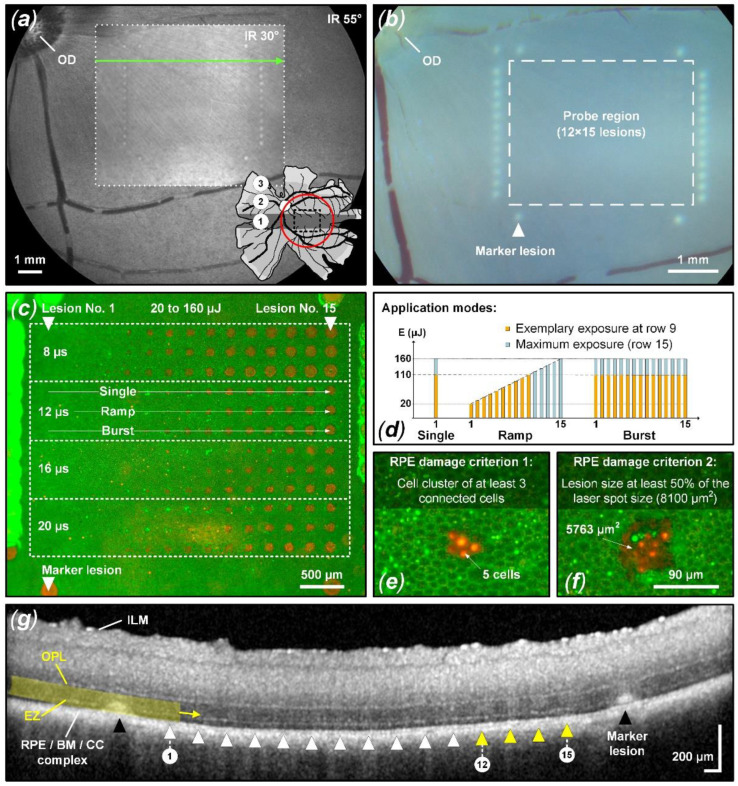
Experiment overview based on sample No. 3. (**a**) IR cSLO image (33° and 55°) of the pig fundus. The pig retina map shows the IR cSLO 55° FOV (red circle) and the three main retinal regions: the visual streak (VS, No. 1: black), the mid-periphery (No. 2: gray), and the periphery (No. 3: pale gray) [41]. The treatment pattern was placed within the VS close to the optic disc (OD). (**b**) Corresponding CFP image showing marker lesions (white triangle) around the probe region (white-dashed rectangle; 12 × 15 lesions) where the microsecond treatment pattern was applied. (**c**) Complementary live/dead RPE cell viability assay showing the treatment pattern with four different probe regions for pulses of 8, 12, 16, and 20 µs duration, each containing three different application modes (single, ramp, burst (**d**)). (**e**,**f**) Close-up view of individual lesions showing the two RPE damage assessment criteria. Green-fluorescent calcein-AM (live) and red-fluorescent ethidium homodimer-1 (EthD-1, dead) as well as hyperfluorescence indicate function or loss of plasma membrane integrity. (**g**) OCT B-scan of the 8 µs single pulse probe region (scan position and direction indicated as a green arrow in (**a**)). Possible retinal perturbation was assessed from RPE/BM/CC complex up to the OPL with a focus on the EZ. Lesions 12 through 15 show barely visible hyperreflectivity in the EZ region, indicating mild suprathreshold treatment laser interaction.

As depicted in Figure 1a, the porcine retina has, in addition to a mid- and peripheral region, a rod-enriched periphery and a cone-enriched area centralis, also referred to as a visual streak (VS), that resembles the human macula [39,40,41]. The treatment for the experiment presented here was always applied within the VS area. To reach the VS area, a wide-angle lens (Widefield Imaging Module, Heidelberg Engineering, Heidelberg, Germany) with a 55° field of view was used in front of the cSLO. Once the desired area was localized (VS area nasal to the optic disc), the delivery optics were switched back to the standard 30° lens for the remaining treatment.

The treatment pattern consisted of a test region surrounded by 29 reliably detectable marker lesions (Figure 1b). These marker lesions were applied in CW mode (200 ms pulse duration and 200 mW pulse power). Inside the demarcation frame, the microsecond laser probe region was placed, consisting of a pattern of 12 × 15 lesions. Sets of laser pulses with increasing energy grouped in durations of 8, 12, 16, and 20 µs were applied. The pulse energy was adapted according to a recent in vivo animal study [22,42] to meet the microsecond pulse duration range described in detail in the introduction. As depicted in Figure 1c, the pulse duration was increased from top to bottom and the pulse energy was increased from left (20 µJ) to right (160 µJ).

Each pulse duration setting was further split into three different laser application modes: single pulse, pulse ramp (1 to 15 pulses, 100 Hz repetition rate) and pulse burst (15 pulses, 100 Hz repetition rate). Thereby, the individual maximum pulse energy per lesion was identical within vertical subgroups, while the accumulated exposure differed horizontally. The pulse energy was measured using a calibrated energy meter (J-10MB-LE, Coherent, Santa Clara, CA, USA) prior to the experiments in front of the laser aperture of the system.

In each case, the exposure position of the retina was optimized using live OCT B-scans prior to laser application. This ensures that each lesion is optimally focused despite chromatic aberrations.

### 2.4. Fundus Examinations

Within 30 min after laser irradiation, color fundus photography (CFP) and OCT B-scans were acquired. CFP was assessed using a fundus camera (Fundus Module 300, Haag-Streit, Köniz, Switzerland). OCT B-scans were acquired over the treatment region using a pre-defined OCT scan pattern (size: 30° × 20° (8.9 × 6.0 mm^2^); number of B-scans: 97; images averaged per B-scan: 38) to compare morphological changes with RPE lesion formation.

For probit analysis, barely visible and visible lesions on CFP imaging were scored as 1 (suprathreshold). Lesions not visible according to CFP were scored as 0 (subthreshold). Using the OCT B-scans possible retinal perturbation was assessed with emphasis on the ellipsoid zone (EZ), formerly known as the inner/outer segment of photoreceptors (IS/OS), and the area up to the outer plexiform layer (OPL) (Figure 1g). Suprathreshold treatment (scored as 1) was considered to have occurred when any of the following changes were evident within a B-scan in the laser lesion: thickening and hyperreflectivity in the EZ region and outer nuclear layer (ONL), continuous columnar hyperreflectivity from the RPE/BM (Bruch’s membrane) complex to the OPL, external limiting membrane (ELM) upward protrusion and disturbed layer continuity. Damage would of course also have been assessed if the choroid or the entire retina up to the ILM would indicate structural alterations to the undisturbed surroundings. Figure 1g depicts an example of suprathreshold retinal tissue alterations (yellow triangles and marker lesions). Lesions without the previously described tissue alterations were considered subthreshold and scored as 0.

### 2.5. RPE Cell Viability Assay

After microsecond laser irradiation, the viability of RPE cells was tested by using a two-color assay live/dead staining kit (L3224, Thermo Fisher Scientific, Waltham, MA, USA). The eye was carefully cut in half and the vitreous body was removed. Subsequently, a circular area on the retina was excised around the pattern with the help of the visible marker lesions. Successively, the specimens were placed in phosphate-buffered saline (PBS) for 30 min. This gently detaches the retina from the RPE and facilitates complete retinal removal. The specimens were further incubated for 30 min at room temperature using the staining kit. The viable-lethal analysis was conducted using a fluorescence microscope (Axio Lab.A1, Carl Zeiss, Oberkochen, Germany) and attached microscope camera (Gryphax Progres, Jenoptik, Jena, Germany). Thereby, green-fluorescent (emission: 517 nm) calcein-AM (live) and red-fluorescent (emission: 617 nm) ethidium homodimer-1 (EthD-1, dead) as well as bright green hyperfluorescence indicate function or loss of plasma membrane integrity. Post processing of the fluorescence images was accomplished with the Fiji image processing package distribution of ImageJ [43].

For probit analysis, successful damage to the RPE was assessed in binary fashion using two criteria: pronounced lesion area/three cell clusters. For the criterion defined as “area,” the lesion area (including hyperfluorescent cells) had to exceed or fall below 50% of the square beam profile of 90 × 90 μm^2^ and thus be scored as 1 (RPE damage present) or 0 (no damage). For the criterion defined as “cluster”, three contiguous dead RPE cells (including hyperfluorescent cells) had to be present within the treatment spot area.

### 2.6. Fringe Washout Evaluation in OCT M-Scans

As previously mentioned, the Spectralis Centaurus device can be operated in M-scan mode to measure time-resolved sequences of A-scans at the therapy laser application site before, during and after the exposure. The evaluation of SD-OCT M-scans for fringe washouts was performed post-treatment visually (investigator based) and using the algorithm for RFD introduced by Burri et al. [31].

In view of future clinical applications, the RFD algorithm features a variable parameter (κ-value). This parameter determines the sensitivity ratio between the fringe washout-based filter edge response and the background noise of the M-scan and has to be set manually by the operator. This will give the dosimetry algorithm a certain flexibility to adapt to different anatomical, optical or clinical ocular conditions. To check which algorithm setting could be suitable for future clinical applications, M-scan data was analyzed for fringe washouts with different κ-values. Therefore, all acquired M-scans were evaluated with 18 different κ-values ranging from 5 to 100.

For probit analysis, fringe washout, i.e., loss of the interference signal was defined as a temporal signal loss over one A-scan integration time (11.8 µs) or longer and axial over 5 pixels (11.4 µm/pixel) or more as a criterion for the investigator-based examination. Such a signal loss was scored as 1 (fringe washout present). Weaker signal losses or signal disturbances (change in scatter behavior) were not evaluated and scored as 0 (no fringe washout). For the algorithm, probit analysis was performed based on the detected fringe washouts according to the overall best-performing κ-values (single: κ=18; ramp: κ=11; burst: κ=12).

### 2.7. Probit Analysis

To investigate the influence of different laser parameters a probit analysis was performed to calculate dose–response percentiles. The median exposure dose, i.e., the dose at which 50% of the exposures resulted in a response, i.e., a detectable RPE laser lesion, is referred to as the effective dose ED_50_. Correspondingly, the exposure doses at which 16% and 84% of the exposures resulted in detected lesions are referred to as ED_16_ and ED_84_, respectively. The logarithms of these points represent 1 standard deviation in the normal distribution from the logarithm of the median dose [44]. The previously mentioned binary evaluation for RPE cell damage, fringe washouts in OCT M-scans as well as suprathreshold lesions according to CFP and OCT B-scan imaging served as input for the probit analysis. The analysis was performed with Origin 2023 (OriginLab Corporation, Northampton, MA, USA) utilizing the Levenberg Marquardt iteration algorithm to fit with a χ^2^ tolerance value of 1 × 10^−9^ within up to 400 iteration steps.

### 2.8. Statistical Evaluation

The ground truth for the statistical evaluation was based on the RPE cell viability assay and the hypothesis that RPE lesions due to MBF lead to fringe washouts in SD-OCT M-scans. For statistical evaluation, the occurrence of fringe washouts (predictive class) was compared to the damage outcome on the viability assay (actual class) by using the confusion matrix as described by Burri et al. [31]. The confusion matrix features four cardinalities: true positive (TP), true negative (TN), false positive (FP) and false negative (FN). Thereby, positive and negative refer to the prediction (true or false) made by SD-OCT M-scans of whether an RPE lesion was created, based on the presence of fringe washouts. Based on the four cardinalities of the confusion matrix, several statistical measures were derived to present the overall device performance in a straightforward fashion. In our case, the sensitivity, specificity, accuracy, positive predictive value (PPV) and negative predictive value (NPV) were calculated. Statistical measures were then analyzed via a receiver operating characteristic (ROC) curve. The ROC of an algorithm shows its performance as a tradeoff between selectivity and sensitivity, with the optimum performance in the top-left corner of the graph. Therefore, we used the ROC curve to determine the RFD algorithm’s optimal κ-value by checking the optimal operating point (OOP) for each pulse duration and each application mode. To determine for which pulse duration and application mode the dosimetry algorithm gave the best overall performance, the area under the ROC curve (AUC) was calculated.

## 3. Results

### 3.1. RPE Cell Damage Thresholds

**Figure 2:**
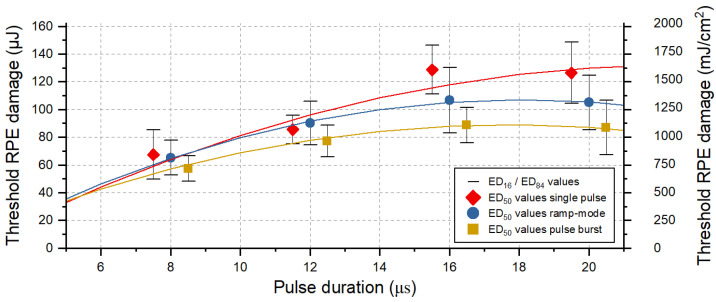
RPE cell damage threshold exposures values for pulses of 8, 12, 16 and 20 µs duration and different application modes: single pulse (red diamond), ramp mode (blue circle) and pulse bursts (orange square). Shown is the evaluation according to the criterion for pronounced lesion, i.e., when the treated area within the 90 × 90 μm^2^ square beam profile exceeded 50% of the lesion size. The error bars around the ED_50_ values indicate the width of the normal distribution (ED_16_ and ED_84_). The data points were fitted using OriginLab’s polynomial fit function, indicated as solid lines.

In total, 1620 laser lesions were applied to the retina of nine ex vivo porcine eyes. This quantity of lesions evaluated via the RPE cell viability assay has proven to be sufficient for probit analysis (χ^2^ tolerance has been reached). Figure 2 summarizes the resulting RPE cell damage threshold values for pulses of 8, 12, 16 and 20 µs duration grouped for application modes (single, ramp and burst), using the evaluation criterion for pronounced RPE lesions (area). The exact threshold values per laser pulse duration and application mode can be found for both RPE damage criteria in Table A1 (cluster) and Table A2 (area) in Appendix A.

Our results indicate that for pulse durations shorter than 12 µs, no cumulative RPE damage effects occur for the laser application in ramp mode, with increasing pulse energy (over maximal 15 pulses) and by using a repetition rate of 100 Hz. This is shown by the comparison of the damage threshold ratios for the different application modes (Figure 2). For pulse durations of 16 µs and 20 µs, however, the damage threshold for pulses applied in ramp mode is lower by a factor of 1.2 (Appendix A: Table A2). A lower RPE damage threshold compared to single pulses is also found in pulse burst mode. Here, a factor of 1.5 is found for pulse durations of 16 µs and 20 µs. The fitted data points indicate that for pulse durations shorter than approximately 6 µs, no or only very limited cumulative RPE damage effects would occur for the application of pulse burst.

**Figure 3:**
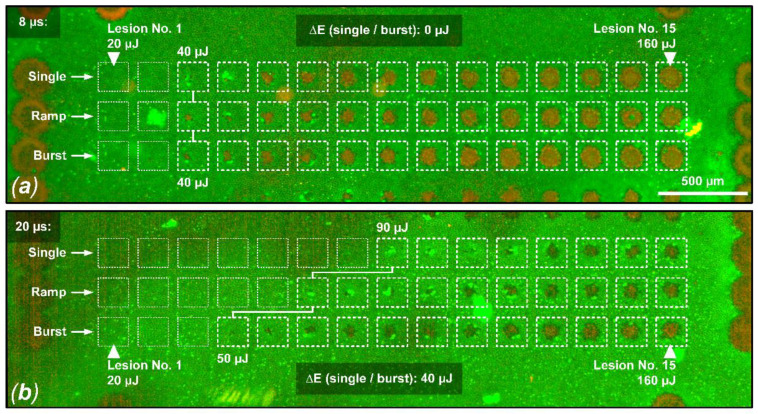
Live/dead RPE cell viability assay for sample No. 8. Shown is a comparison between the probe zone for 8 µs pulses (**a**) and 20 µs pulses (**b**). In this example, the damage criterion was defined as hyperfluorescence or RPE damage in a cell cluster over at least 3 cells in the area of a laser lesion. (**a**) For all three laser application modalities (single, ramp, burst), the same RPE damage threshold value of 40 µJ (494 mJ/cm^2^) can be observed when applying laser pulses of 8 µs duration. (**b**) For the application of laser pulses with a duration of 20 µs, the RPE damage threshold for the pulse burst application (15 pulses, 100 Hz repetition rate) is 40 µJ lower (factor 1.8) than for the single pulse application, indicating cumulative damage effects (single pulse threshold: 90 µJ (1111 mJ/cm^2^), pulse burst threshold: 50 µJ (617 mJ/cm^2^)).

Figure 3 shows an evaluation of the viability of RPE cells for sample No. 8, focusing on pulse durations of 8 µs and 20 µs, and reflects the results described in Figure 2. In this case, no threshold difference for RPE cell damage was found for different application modes (single, ramp and burst) for pulses of 8 µs duration. However, for pulses of 20 µs duration, a threshold ratio of 1.8 was found for the application of a pulse burst compared to the single pulse application.

### 3.2. Results of CFP and OCT B-Scan Imaging

**Figure 4:**
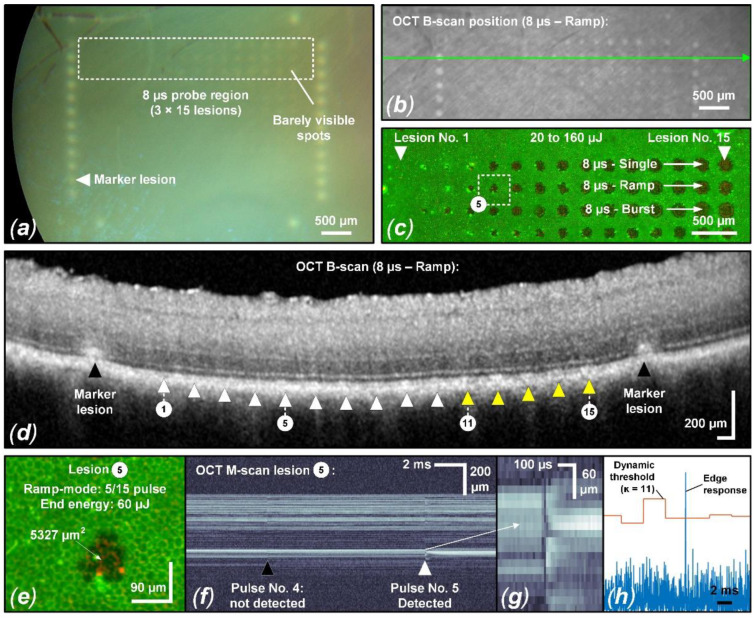
Evaluation of sample No. 6 with suprathreshold laser spots exposing 90 × 90 μm^2^ (**a**) CFP image featuring suprathreshold lesions in the 8 µs sample region. Barely visible lesions appear from 100 µJ (1235 mJ/cm^2^) to 120 µJ (1481 mJ/cm^2^). (**b**) Corresponding IR cSLO image indicating the OCT B-scan position across the 8 µs ramp mode probe region. Matching the CFP image, certain lesions show hyperreflectivity. (**c**) Complementary live/dead RPE cell viability assay of the 8 µs probe region indicates RPE damage starting from 30 µJ (370 mJ/cm^2^) to 50 µJ (617 mJ/cm^2^) (hyperfluorescence/damaged RPE cell clusters). (**d**) OCT B-scan of the 8 µs ramp mode probe region. Lesions 11 through 15 can be associated with barely visible hyperreflectivity of the CFP in the EZ region, indicating mild suprathreshold treatment laser interaction. (**e**) Focus on lesion No. 5 from the 8 µs ramp mode probe region: RFD reliably detected fringe washouts from this lesion onwards (**f**–**h**). Visually, a slight fringe washout is already evident for the fourth pulse.

**Figure 5:**
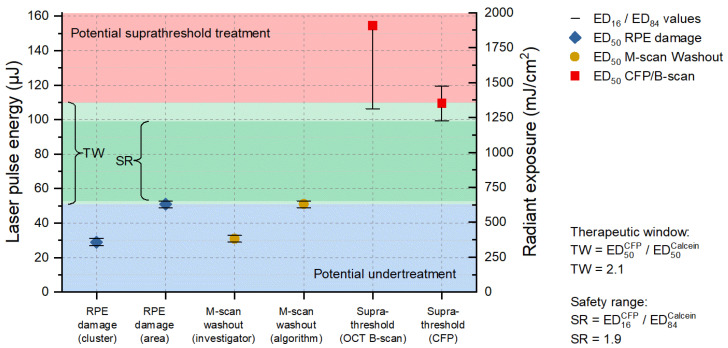
Probit analysis for the 8 µs probe region of sample No. 6. The blue area shows the area for undertreatment (based on the cell viability assay); the red area is the threshold towards suprathreshold lesions (based on the CFP evaluation); and the green area in between the resulting therapeutic window (TW = 2.1); and the dark green a more narrowly defined safety range (SR = 1.9). The blue diamond symbols show the RPE damage threshold (criterion cluster/area); the orange circles the thresholds for detection of fringe washouts in SD-OCT M-scans (investigator based/algorithm); and the red squares the thresholds for suprathreshold effects (according to OCT B-scans/CFP).

Visual CFP evaluation could only detect tissue changes in 2 of 9 samples for pulse durations of 8 µs and 12 µs. Therefore, an accurate overall threshold determination for ophthalmoscopically visible changes and the calculation of the therapeutic window (TW) and its associated safety range (SR) were not possible and would require extending the energy and pulse duration range. Furthermore, an evaluation of suprathreshold lesions using OCT B-scans and the evaluation criteria described in Section 2.4 did not yield clear results. Only a few samples showed barely visible tissue alterations, which made it impossible to perform probit analysis. However, the evaluation shown in Figure 4 depicts the sample in which barely visible tissue whitening was best visible according to CFP, and correlated tissue alterations in OCT B-scans were found. This sample was also found to have the lowest overall threshold for RPE damage (Figure 5), which indicates high ocular transmission and strong RPE pigmentation which probably led to these suprathreshold effects.

As evident in the CFP image (Figure 4a), particularly in the 8 µs probe region, barely visible tissue whitening can be observed, which indicates suprathreshold laser tissue interaction effects. As depicted in Figure 5, we found an ED_50_ value of 109 µJ (1346 mJ/cm^2^) for suprathreshold lesions according to CFP. Such lesions are also visible as hyperreflective lesions in the corresponding IR cSLO image (Figure 4b). The comparison between CFP and cSLO also underlines the principle capability differences between the techniques. While CFP heavily suffers from deterioration of image quality through the neuroretina, caused by higher scattering at shorter wavelengths, the almost twice as long wavelength and the confocal suppression of a significant amount of scattered radiation in cSLO and OCT improve delineation of the lesion borders. OCT furthermore removes axial blur by its significantly more precise coherent depth resolving ability, therefore improving the overall spatial precision. Complementarily, for the barely visible laser lesions according to CFP, the RPE cell viability assay unveils that lesions are significantly larger than the laser spot size of 90 × 90 µm^2^ (8100 µm^2^). For example, the 8 µs single pulse lesion No. 15 applied with a pulse energy of 160 µJ (1975 mJ/cm^2^) was found to have a spot size of approximately 17,800 µm^2^, which is more than twice the applied laser spot size. This increase in laser spot size indicates a lateral (and thus probably also axial) heat diffusion (Figure 4c). The corresponding OCT B-scan images show slight abnormalities in the EZ area for individual lesions applied at high pulse energy. As depicted in Figure 5, we found an ED_50_ value of 154 µJ (1901 mJ/cm^2^) for suprathreshold lesions according to the OCT B-scan imaging. For example, for the 8 µs ramp mode application, morphological changes were observed for lesions No. 11 to 15 (120 µJ (1481 mJ/cm^2^) to 160 μJ (1975 mJ/cm^2^)) (Figure 4d). In the area of the EZ up to the OPL, a slight hyperreflectivity was apparent. Furthermore, at the level of the RPE/BM complex, mild hyperreflectivity and local tissue expansion are partially visible as a kind of RPE clumping. Otherwise, the retinal structure seems unchanged. The EZ as well as the OPL are continuous compared to the marking lesions where their layered structure is disrupted.

Since we know from the results according to Section 3.1 that there are almost no cumulative damage effects for pulses of 8 µs duration, we combined all lesions (single, ramp, burst) of sample No. 6, which proved to be sufficient for probit analysis. Thus, a calculation of the TW and the SR could be performed (Figure 5). The term TW has been introduced in the context of selective RPE laser treatment to describe the available range of permitted irradiance and radiant exposure levels as the ratio between overtreatment utilizing the ophthalmoscopic ED_50_-value and undertreatment via the angiographic ED_50_-value [34,45], based on the statistical expectation that 50% of the treatment sites experience an effect at that effective dose (ED). In our case, RPE damage was determined by the cell viability assay instead of angiography. Complementarily, the SR is evaluated as the ratio of the ophthalmoscopic ED_16_-value and the ED_84_-value for RPE damage, utilizing the standard deviation as a quantitative measure of reliability. Generally, large TW and SR ranges permit successful treatment, even with larger variations of the environment (e.g., absorptance, optical quality, stability of the delivery system). Even if an exposure system can be fine-tuned to match the average local situation within the exposure field, the TW and SR at least have to cover the variations within this field (e.g., absorption variances between cells, irradiance fluctuations due to speckles, image border and heat accumulation effects in the center). The results shown in Figure 5 are not statistically representative due to the sparse dataset on only one sample, but they do provide some indications for the TW and SR using laser pulses of 8 µs duration.

For a pulse duration of 8 µs a TW of 2.1 and a SR of 1.9 was found (Figure 5). Fringe washouts in SD-OCT M-scans were found from pulse energies (ED_50_) higher than 31 µJ (383 mJ/cm^2^) for the investigator-based evaluation and higher than 51 µJ (630 mJ/cm^2^) for the RFD algorithm evaluation. This means that RFD feedback allows us to operate extremely close to the local average RPE damage threshold and utilize the TW almost solely for compensation within the exposure field. For this sample, all RPE lesions were correctly predicted for pulse energies (ED_50_) higher than 62 µJ (765 mJ/cm^2^), onwards, with a sensitivity of 100%. Thus, acting in the lower SR range would have prevented any suprathreshold lesion according to CFP and OCT B-scan imaging in a real-time application. Figure 4e–h shows that, from lesion number 5, applied with a pulse energy of 60 µJ (741 mJ/cm^2^), onwards, SD-OCT M-scan fringe washouts were reliably detected using the RFD algorithm.

### 3.3. Evaluation of Fringe-Washouts in OCT M-Scans for RFD

**Figure 6:**
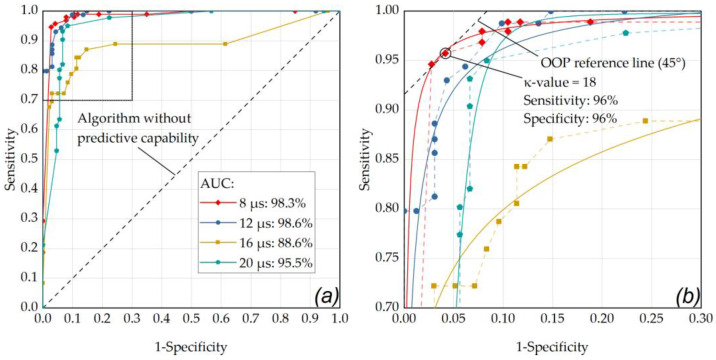
(**a**) ROC curve of the RFD algorithm showing the performance for the single pulse application mode at different pulse durations with respect for different *κ*-values (factor for M-scan washout detection threshold above noise floor) and for the criterion for pronounced RPE lesions (area) in the cell viability assay. (**a**) The best performance was obtained for a pulse duration of 12 µs (AUC: 0.986) and 8 µs (AUC: 0.983). (**b**) enlarged ROC section (sensitivity 0.7 to 1, 1-specificity from 0 to 0.3): the data point that is closest to the 45° OOP reference line was determined as *κ*-value of 18 at a laser pulse duration of 8 µs (sensitivity and specificity of 96%). The diagonal line in these graphs represents an algorithm without predictive capability. The data points were fitted using OriginLab’s Logistic function (Logistic-FitFunc, indicated as solid lines).

Fringe washouts in SD-OCT M-scans were evaluated visually (investigator based) and using the RFD algorithm (an example of a fringe washout is shown in Figure 4f,g). Thereby, different *κ*-value settings ranging from 5 to 100 were used with the RFD algorithm and compared to levels of RPE cell damage. The resulting statistical measures (sensitivity and specificity) are described by the ROC curve in Figure 6. The evaluation shows the results for the evaluation of single pulses and using the criterion for pronounced RPE lesions (area) in the cell viability assay.

**Figure A1:**
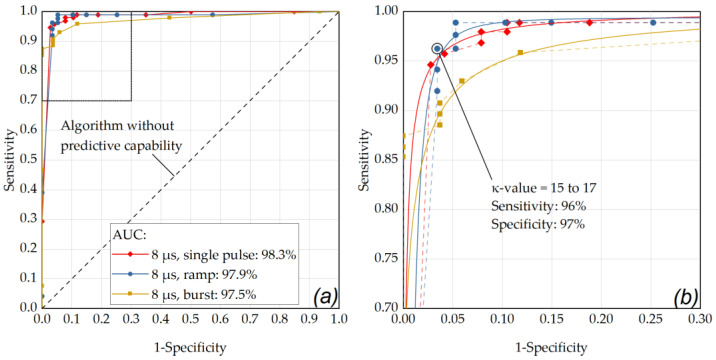
(**a**) ROC curve of the RFD algorithm showing the performance for pulses of 8 µs duration and the different application modes (single, ramp, burst) with respect for different *κ*-values and for the criterion for pronounced RPE lesions (area) in the cell viability assay. The best performance was obtained for the single pulse application (AUC = 0.983). An almost similar performance was achieved for the ramp (AUC = 0.979) and burst application (0.975). (**b**) Enlarged ROC section (sensitivity 0.7 to 1, 1-specificity from 0 to 0.3): the data point that is closest to the 45° OOP reference line was determined as *κ*-value of 15 to 17 for the ramp mode application (sensitivity: 0.96, specificity: 0.97).

**Figure A2:**
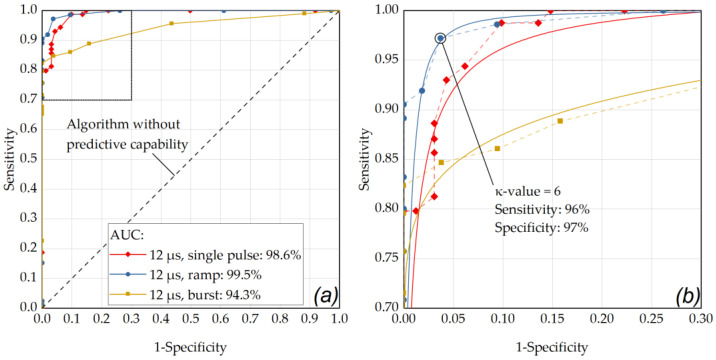
(**a**) ROC curve for a pulse duration of 12 µs. The best performance was obtained for the ramp mode application (AUC = 0.995). (**b**) The OOP was determined as *κ*-value of 6 for the ramp mode application (sensitivity: 0.96, specificity: 0.97).

As depicted in Figure 6, the optimal operating point for the application of single pulses was found for a laser pulse duration of 8 µs (sensitivity: 96%, specificity: 96%, AUC: 0.983) using a *κ*-value of 18. Appendix B contains further ROC analyses with comparisons within the individual pulse durations for the different application modes. As depicted in Figure A1, by using pulses of 8 µs duration in ramp mode and an RFD algorithm sensitivity setting in the range of *κ* = 15 to *κ* = 17, a sensitivity of 96% and a specificity of 97% were achieved (AUC: 0.979). Figure A2 shows that similar results were obtained by using pulses of 12 µs duration in ramp mode and an RFD algorithm sensitivity setting of *κ* = 6 (sensitivity: 96%, specificity: 97%, AUC: 0.995).

Across all samples, a median *κ*-value of 12 was found to work best for the different pulse durations and application modes. As shown in Table A3, for this setting, the RFD algorithm achieved an overall accuracy of 93% (sensitivity: 91%, specificity: 94%). The highest accuracy (97%) was achieved for a pulse duration of 8 µs in ramp mode. In this case, a sensitivity of 99% and a specificity of 93% were obtained. The lowest accuracy (89%) was achieved for single pulse application with a pulse duration of 16 µs and burst application with a pulse duration of 20 µs. In both cases, a sensitivity of 85% and a specificity of 94% were obtained.

In addition to the RFD algorithm-based evaluation, Table A4 in Appendix C shows the statistical evaluation for the investigator-based fringe washout evaluation in SD-OCT M-scans. For this evaluation, the highest accuracy (97%) was achieved for single pulse applications using pulses of 8 µs duration (sensitivity: 98%, specificity: 90%). The lowest accuracy (50%) was found for single pulses of 16 µs pulse duration (sensitivity: 100%, specificity: 34%). A similarly low accuracy was also measured for 20 µs single pulse applications (accuracy: 54%, sensitivity: 100%, specificity: 38%).

## 4. Discussion

As already found in in vivo experiments by Framme et al. in 2004, when using laser pulses of 200 ns and 1.7 µs duration, the laser pulse duration and the number of applied pulses have a significant influence on RPE damage thresholds and consecutively on TW and SR [46]. In this work, we have investigated a range of longer, better controllable pulse durations utilizing a flexible high-power CW prototype SRT laser in the range of 8 µs to 20 µs with different application modes (single, ramp, burst), with a precisely positionable exposure field and a sensitive set of diagnostic methods to determine cellular effects. Recently, a Q-switched Nd:YLF (Neodymium-doped Yttrium Lithium Fluoride) laser emitting at 527 nm has been widely used for SRT. Since this laser operates at a pulse duration of 1.7 µs, relatively high powers around 200 W are required to achieve the desired maximum pulse energy of up to 350 µJ [47]. However, our results indicate that, especially for pulse durations shorter than 12 µs, new flexible CW lasers with high power (around 30 W) should also be considered for SRT, since at these pulse durations RPE damage seems to be possible without cumulative damage. In other words, with the laser used for this study and under suitable boundary conditions such as a repetition rate of 100 Hz, it is apparently possible to work within the thermal confinement of the RPE cell layer. Therefore, this laser has the potential to cover the entire laser treatment range from SRT to sublethal hyperthermia-inducing short pulse applications to traditional CW photocoagulation due to its functionality, flexibility and power.

As already shown in previous work [31] and confirmed by the results of Framme et al. [34,46], this study shows once more that the RPE damage threshold decreases with a shorter pulse duration (Figure 2). With shorter pulses, less heat dissipates from the melanosomes into the tissue. Therefore, less energy is required to heat the melanosomes leading to MBF and consequent rupture of RPE cells [48].

As shown in Figure 2 and the cell viability assay for sample No. 8. in Figure 3, for pulse durations of 16 µs and especially 20 µs, the thermal confinement of the RPE seems to be exceeded. Cumulative RPE damage effects become evident when multiple pulses per lesion are delivered at a repetition rate of 100 Hz for these pulse durations. The tissue has too little time to sufficiently cool during the individual pulses, which probably leads to a slow but steady increase in the RPE’s baseline temperature. Therefore, if heat diffusion into the neuronal retina and especially to the photoreceptors is to be prevented, as in the sense of SRT, these pulse durations seem rather unsuitable considering the applied repetition rate of 100 Hz. For pulse durations of 12 µs and especially of 8 µs, only very small cumulative effects appear. In particular, the application of pulse ramps, which is common for SRT to figure out the local TW, seems to be unproblematic. This observed transition around 12 µs is also in good agreement with previous model calculations by Brinkmann et al. who estimated a thermal relaxation time of 10 µs for RPE cells [3]. Furthermore, Schuele et al. were able to show that using laser pulses of 5 µs duration RPE cell damage was always associated with MBF, whereas at pulses of 50 µs duration, thermal denaturation takes over but does not fully replace microbubble formation [49]. Complementarily, Lee et al. were able to show that by using pulses of 1, 5 and 10 µs duration, more than 95% of dead RPE cells were associated with MBF, whereas the ratio decreased to 65% and 45% for longer 20 µs and 40 µs pulses, respectively [30]. The presented results from this study underline these findings.

Using probit analysis, we were able to determine the TW and SR for a pulse duration of 8 µs from one sample. We found a TW of 2.1 and an SR of 1.9. Similar results were found by Framme et al. in an in vivo rabbit model using a laser emitting at 527 nm and pulses of 1.7 µs duration [46]. For the application of single pulses and pulse burst with 10 pulses (repetition rate: 100 Hz), they found a TW of 1.6 and 2.5, respectively. For the application of pulse bursts containing 10 and 100 pulses, they found an SR of 1.0 and 1.7, respectively. As mentioned before, our results are statistically not representative due to the sparse data set from a single sample. Nevertheless, our result fits well into the results from Framme et al. and thus provide the first indications on the TW and SR when using laser pulses with a duration of 8 µs. Further experiments are certainly necessary to define the TW and the SR for SRT at the pulse duration used by us in order to ensure a safe clinical application.

CFP unveiled only a limited number of barely visible lesions within the SRT probe areas, which indicate marginally suprathreshold treatments. Such lesions are also described in a clinical SRT study by Park et al. and were not defined as conventional coagulation scars as they differ in shape, color and appearance [14]. Clinically, such lesions did not lead to long-term morphological changes in the retina and disappeared within six months. The same applies to the corresponding slight morphological changes according to the OCT B-scan images. Such effects are also known from clinical SRT studies and were reversible without retinal damage [50]. However, an improved RFD algorithm should avoid marginal suprathreshold effects in the first place.

Recently, similar laser parameters were applied to porcine RPE–choroid–sclera explants using the Spectralis Centaurus device [31]. In these experiments, we found a similar setting (*κ*-value of 13 at a laser pulse duration of 6 µs) as the optimal operation point for the RFD algorithm. However, the achieved specificity (94%) and especially the sensitivity (89%) at that time were considerably lower than in the experiments shown here. With hindsight, we can see that this was caused by the utilized RPE–choroid–sclera explants and the RPE cell viability assay evaluation criterion of small cell clusters. On RPE–sclera–choroid explants, our RFD algorithm has a much smaller measurement range for detecting fringe washouts in SD-OCT M-scans due to the absence of the neurosensory retina. This difference resulted in a higher sensitivity of up to 96% (Figure A1 and Figure A2) in this experiment. Since whole eyes would of course be treated in a clinical setting, it can be assumed that the high sensitivity of around 96% would also be achieved in humans. Even more so, it can be assumed that in humans the OCT feedback would further improve with an optimally moistened cornea and a retina free of edema based on the in vivo situation. Furthermore, the RFD algorithm was developed for a different positive detection criterion (at least 50% dead RPE cells within the exposure spot) than the criterion used in the last experiment (cluster of three dead RPE cells). This criterion led to fine fringe washouts being disregarded by the algorithm, which resulted in a low number of falsely identified events and therefore a slightly lower specificity. Therefore, as suggested in the last paper, an area-based detection criterion (at least 50% dead RPE cells within the exposure spot) was implemented [31]. This criterion is more appropriate for SRT application since it can be assumed that for the desired stimulation of metabolism after RPE cell proliferation and migration, a certain area of dead RPE cells must be present, and single dead RPE cells are not purposeful. Generally, the RFD algorithm shows a very high overall accuracy of 93% (sensitivity: 91%, specificity: 94%) when predicting RPE lesions. In particular, in the regular SRT application mode using pulse ramps, with a pulse duration of 8 µs, the performance (AUC: 98%) and the accuracy of 97% (sensitivity: 99%, specificity: 93%) are remarkable. This setting has already emerged in previous experiments [19,22,31,33,42] and is, therefore, currently also being evaluated clinically (ClinicalTrials.gov Identifier: NCT04968756). Therefore, in vivo RFD data on our SD-OCT M-scan based algorithm and the Spectralis Centaurus device should be available soon.

The investigator-based evaluation of fringe washouts in SD-OCT M-scans yielded better results for the prediction of RPE cell clusters than for pronounced lesions (Table A4). For example, for a pulse duration of 12 µs and application in ramp mode, an accuracy of 96% (sensitivity: 97%, specificity: 90%) was obtained for RPE cell clusters, while an accuracy of 73% (sensitivity: 100%, specificity: 45%) was obtained for the detection of pronounced RPE lesions. The reason for this is that visual signal changes in the SD-OCT M-scan (change in scattering behavior) are relatively easy to detect at the level of the RPE cell layer (Figure 4f/Pulse No. 4). These weak signal changes correlate with the destruction of single RPE cells in the laser spot, as we have already shown in previous work [31]. In this respect, the poor specificity of 45% for the detection of pronounced lesions is expected since such lesions are not considered to be lesions anyway. In this case (12 µs, ramp mode), across all samples, 36 of 135 lesions were scored as false positives, and a sensitivity of 100% was achieved across all samples since no false negative evaluation occurred. Although our experiment shows that very good results can be obtained with the RFD algorithm, the above-described circumstance shows the great potential of RFD for SRT using SD-OCT. High sensitivity is mandatory for safe SRT application if suprathreshold lesions are to be prevented. Since our RFD algorithm was optimized to detect signal loss over one A-scan integration time rather than slight signal changes, we still see great potential to further optimize our RFD algorithm.

We found different optimal settings (individual *κ*-values) for the samples. Thus, for certain samples and a median *κ*-value of 12, we partially found an accuracy, sensitivity and specificity of 100%. For other samples, however, this value works less optimally. In this respect, it could be important to individually adapt the optimal settings when using the RFD algorithm in patients. If necessary, the baseline noise level could be re-determined or re-measured at each treatment site.

## 5. Limitations

A limitation of our experiment is of course related to the ex vivo experimental setting. Although the eyes were treated as soon as possible after enucleation, the intraocular pressure was maintained and the cornea was moistened, the thermodynamic processes, i.e., the movement of heat energy, may have been different from an in vivo situation. Temperature rise in tissues depends on many different factors such as heat conduction into the tissue, metabolic heat generation, and heating (cooling) by blood circulation in the tissue. For example, in the ex vivo situation, blood circulation no longer takes place and also the baseline temperature of the eyes in our experiment was at room temperature and not body temperature. Thus, there is a different heat flow or possibly also a heat accumulation in the retina that would take place differently in vivo. In this respect, the data obtained here cannot replace in vivo experiments, but they show a certain tendency about the laser–tissue interaction that could also take place in vivo. For example, in an in vivo experiment with pigmented rabbit eyes, we have already shown that when laser pulses of 20 µs duration are applied in ramp mode, more pronounced lesions appear according to OCT B-scans compared to pulses of 4 or 8 µs duration [22]. This circumstance is reflected in this ex vivo experiment, which is why it can be assumed that the present results may also be representative of the in vivo situation.

Another limitation, which is also due to the ex vivo setting, was the OCT quality. In the OCT-B scans shown here, it is evident that despite the short time between enucleation and treatement, the retina had already begun to retain water (edema formation). As a result, the retinal structures were significantly worse to assess than one would expect from an in vivo situation. Therefore, morphologic changes were difficult to detect in OCT B-scans. Furthermore, this circumstance has a negative impact on the performance of the RFD algorithm, since a good signal-to-noise ratio (SNR) is important to reliably detect fringe washouts in OCT M-scans. In this respect, even better OCT dosimetry feedback can be expected in an in vivo setting.

## 6. Conclusions

Our experiments show that microsecond laser pulses shorter than 12 µs appear safe since no cumulative RPE damage effects occur for the ramp mode application mode, which is required in SRT to determine the optimal local exposure settings. However, for pulse durations of 16 µs and 20 µs, cumulative RPE damage effects were observed, especially for pulse bursts. These longer pulse durations, therefore, are less suitable for SRT or require extremely precise control using RFD to prevent suprathreshold effects within the neurosensory retina and especially the photoreceptors. Using our RFD algorithm, which is based on the detection of fringe washouts in SD-OCT M-scans during the application of microsecond laser pulses, RPE lesions were predicted with a high overall accuracy of 93%. The highest accuracy (97%) was achieved for the application of a pulse duration of 8 µs in ramp mode. In this case, a sensitivity of 99% and a specificity of 93% were obtained, which makes this type of RFD algorithm particularly suitable for SRT. However, future research still must verify whether real-time OCT dosimetry in patients undergoing SRT is capable of automatically controlling the level of treatment to induce RPE regeneration without any adverse effects on neighboring tissue. While the focus of the current study was specific to RPE damage, this experiment also shows that SD-OCT M-scan-based RFD, which still functions with high accuracy even at longer pulse durations, could possibly control hyperthermia in the RPE and, thus, be considered as possible dosimetry for subthreshold laser applications.

## Figures and Tables

**Figure A3:**
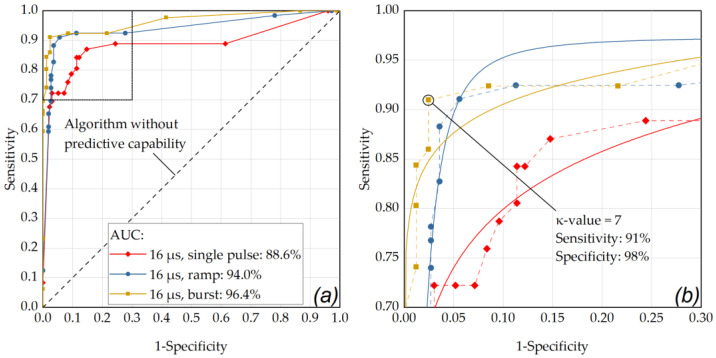
(**a**) ROC curve for a pulse duration of 16 µs. The best performance was obtained for the pulse burst application (AUC = 0.964). (**b**) The OOP was determined as *κ*-value of 7 for the pulse burst application (sensitivity: 0.91, specificity: 0.98).

**Figure A4:**
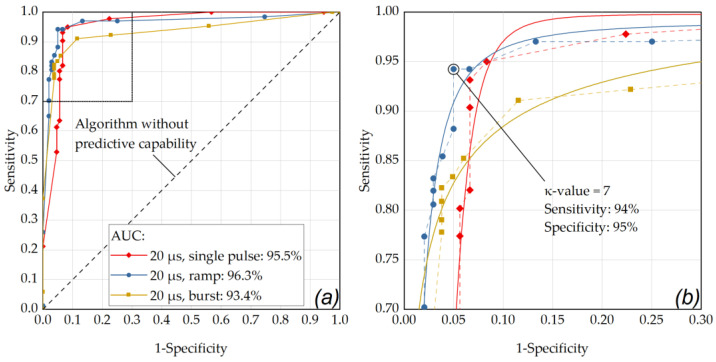
(**a**) ROC curve for a pulse duration of 20 µs. The best performance was obtained for the ramp mode application (AUC = 0.963). (**b**) The OOP was determined as *κ*-value of 7 for the pulse burst application (sensitivity: 0.94, specificity: 0.95).

## Data Availability

The data presented in this study (excluding the SD-OCT dosimetry algorithm) are available on request from the corresponding author.

## Appendix A

The viability of RPE cells was tested by using a two-color assay live/dead staining kit. For probit analysis, successful damage to the RPE was assessed in a binary fashion using two different criteria (pronounced lesions/three cell clusters). For the criterion defined as “area,” the treated area within the square beam profile of 90 × 90 μm^2^ had to exceed or fall below 50% of the lesion size (including hyperfluorescent cells) and thus be scored as 1 (RPE damage present) or 0 (no damage). For the criterion defined as “cluster” on the other hand, three contiguous dead RPE cells (including hyperfluorescent cells) had to be present within the treatment spot area. The exact RPE damage thresholds for both evaluation criteria are listed below.

**Table A1:** RPE damage threshold values (ED values in µJ) for the evaluation criterion “cluster”.

PD	Application Mode	${ED}_{16}^{RPE}$	${ED}_{50}^{RPE}$	${ED}_{84}^{RPE}$	Threshold Ratios (${ED}_{50}$)	
					Single/Ramp	Single/Burst
8 µs	single	28	37	46	0.9	1.2
	ramp	31	39	48		
	burst	20	30	40		
12 µs	single	35	46	56	1.0	1.0
	ramp	41	48	55		
	burst	32	44	57		
16 µs	single	56	72	87	1.1	1.3
	ramp	54	67	79		
	burst	45	54	63		
20 µs	single	61	66	72	1.1	1.3
	ramp	54	60	66		
	burst	40	50	60		

PD: pulse duration; ED: dose–response percentiles; RPE: retinal pigment epithelium.

**Table A2:** RPE damage threshold values (ED values in µJ) for the evaluation criterion “area”.

PD	Application Mode	${ED}_{16}^{RPE}$	${ED}_{50}^{RPE}$	${ED}_{84}^{RPE}$	Threshold Ratios (${ED}_{50}$)	
					Single/Ramp	Single/Burst
8 µs	single	50	67	85	1.0	1.2
	ramp	53	65	78		
	burst	48	57	67		
12 µs	single	75	86	96	1.0	1.1
	ramp	74	90	107		
	burst	66	77	89		
16 µs	single	111	129	146	1.2	1.5
	ramp	83	107	130		
	burst	76	89	101		
20 µs	single	104	126	148	1.2	1.5
	ramp	85	105	125		
	burst	67	87	106		

PD: pulse duration; ED: dose–response percentiles; RPE: retinal pigment epithelium.

## Appendix B

RFD algorithm evaluation for different *κ*-values ranging from 5 to 100 and compared to levels of RPE cell damage. The resulting statistical measures (sensitivity and specificity) are described by the following ROC curves (Figure A1, Figure A2, Figure A3 and Figure A4). The evaluations are divided according to the four different pulse durations. Figure A1 shows the results for a pulse duration of 8 µs, Figure A2 those for 12 µs, Figure A3 those for 16 µs and Figure A4 those for 20 µs. Furthermore, Table A3 shows the values of the statistical analysis for the RFD-based evaluation using the median *κ*-value of 12, which was found to work best for the different pulse durations and application modes over all probes.

**Table A3:** RFD-based fringe washout SD-OCT M-scan analysis using the median *κ*-value of 12.

PD	Mode	TP	TN	FP	FN	PPV	NPV	Sens.	Spec.	Acc.
8 µs	Single	86	43	5	1	0.95	0.98	0.99	0.90	0.96
	Ramp	88	43	3	1	0.97	0.98	0.99	0.93	0.97
	Burst	89	36	1	9	0.99	0.80	0.91	0.97	0.93
12 µs	Single	68	59	6	2	0.92	0.97	0.97	0.91	0.94
	Ramp	63	65	1	6	0.98	0.92	0.91	0.98	0.95
	Burst	68	53	2	12	0.97	0.82	0.85	0.96	0.90
16 µs	Single	29	91	10	5	0.74	0.95	0.85	0.90	0.89
	Ramp	45	80	4	6	0.92	0.93	0.88	0.95	0.93
	Burst	60	64	2	9	0.97	0.88	0.87	0.97	0.92
20 µs	Single	32	92	6	5	0.84	0.95	0.86	0.94	0.92
	Ramp	52	76	4	3	0.93	0.96	0.95	0.95	0.95
	Burst	60	60	4	11	0.94	0.85	0.85	0.94	0.89
Overall		740	762	48	70	0.94	0.92	0.91	0.94	0.93

PD: pulse duration; TP: true positive; TN: true negative; FP: false positive; FN: false negative; PPV: positive predictive value; NPV: negative predictive value.

## Appendix C

Fringe washouts in SD-OCT M-scans were evaluated visually (investigator based) and compared to the two different RPE cell viability assay criteria (pronounced lesions/three cell clusters). Table A4 shows the values of the statistical analysis.

**Table A4:** Investigator-based fringe washout SD-OCT M-scan analysis.

PD	Mode	RPE Eval.	TP	TN	FP	FN	PPV	NPV	Sens.	Spec.	Acc.
8 µs	Single	Cluster	113	18	2	2	0.98	0.90	0.98	0.90	0.97
		Area	86	20	29	0	0.75	1.00	1.00	0.41	0.79
	Ramp	Cluster	107	20	1	7	0.99	0.74	0.94	0.95	0.94
		Area	89	27	19	0	0.82	1.00	1.00	0.59	0.86
	Burst	Cluster	114	13	1	7	0.99	0.65	0.94	0.93	0.94
		Area	98	20	17	0	0.85	1.00	1.00	0.54	0.87
12 µs	Single	Cluster	104	23	5	3	0.95	0.88	0.97	0.82	0.94
		Area	69	26	40	0	0.63	1.00	1.00	0.39	0.70
	Ramp	Cluster	102	27	3	3	0.97	0.90	0.97	0.90	0.96
		Area	68	30	37	0	0.65	1.00	1.00	0.45	0.73
	Burst	Cluster	107	23	3	2	0.97	0.92	0.98	0.88	0.96
		Area	80	25	30	0	0.73	1.00	1.00	0.45	0.78
16 µs	Single	Cluster	84	34	16	1	0.84	0.97	0.99	0.68	0.87
		Area	33	35	67	0	0.33	1.00	1.00	0.34	0.50
	Ramp	Cluster	84	31	17	3	0.83	0.91	0.97	0.65	0.85
		Area	51	34	50	0	0.50	1.00	1.00	0.40	0.63
	Burst	Cluster	98	25	12	0	0.89	1.00	1.00	0.68	0.91
		Area	69	25	41	0	0.63	1.00	1.00	0.38	0.70
20 µs	Single	Cluster	84	35	13	3	0.87	0.92	0.97	0.73	0.88
		Area	35	38	62	0	0.36	1.00	1.00	0.38	0.54
	Ramp	Cluster	90	33	7	5	0.93	0.87	0.95	0.83	0.91
		Area	54	38	43	0	0.56	1.00	1.00	0.47	0.68
	Burst	Cluster	95	26	11	3	0.90	0.90	0.97	0.70	0.90
		Area	70	29	36	0	0.66	1.00	1.00	0.45	0.73

PD: pulse duration; RPE: retinal pigment epithelium; TP: true positive; TN: true negative; FP: false positive; FN: false negative; PPV: positive predictive value; NPV: negative predictive value.
